# People Count: Contact-Tracing Apps and Public Health

**DOI:** 10.3201/eid2709.210754

**Published:** 2021-09

**Authors:** Isaac Chun-Hai Fung, Benedict S.B. Chan

**Affiliations:** Georgia Southern University, Statesboro, Georgia, USA (I.C.-H. Fung);; Hong Kong Baptist University, Hong Kong, China (B.S.B. Chan)

**Keywords:** contact tracing, coronavirus disease, COVID-19, privacy

In *People Count: Contact-Tracing Apps and Public Health*, computer scientist Susan Landau advocates for a public discussion on using contact-tracing applications (apps) in public health (Figure). Landau puts her arguments in a succinct, easy-to-read narrative in 6 chapters.

**Figure:**
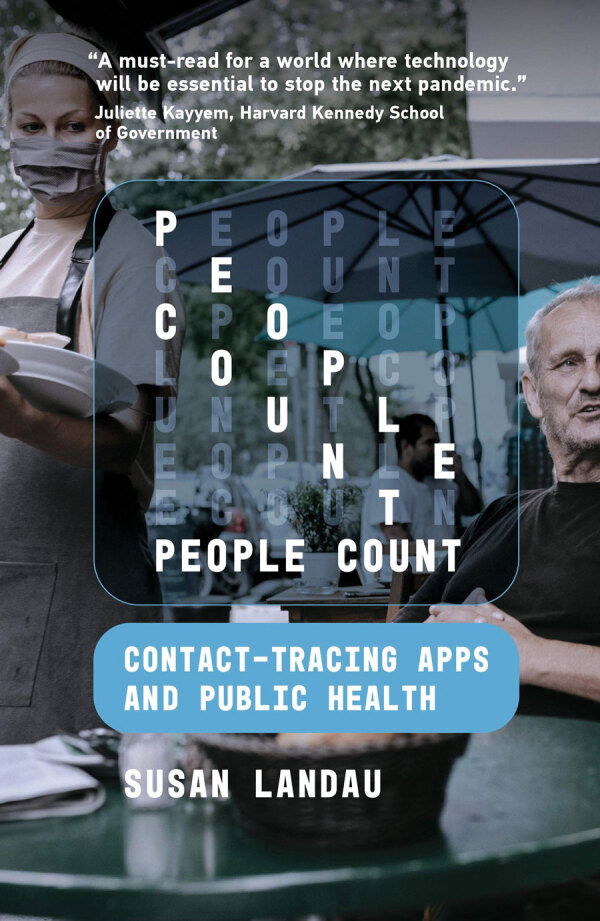
People Count: Contact-Tracing Apps and Public Health

Chapter 1 sets the scene for contact tracing by introducing the basics of epidemiology. Through examples, Chapter 2 explains the implementation of contact tracing and that, for it to succeed, governments must earn the public’s trust by maintaining confidentiality and engaging with communities.

Chapter 3 introduces smartphone technologies proposed to add to contact tracing, focusing on apps with centralized databases, such as Singapore’s TraceTogether, which exchanges identifiers with other users through Bluetooth Low Energy technology. Users authorize the government to view all the information collected from the app. Chapter 4 introduces coronavirus disease (COVID-19) exposure-notification apps, including SwissCovid and COVID Tracker Ireland, which are based on Google Apple Exposure Notification (GAEN) system. Landau raises cybersecurity issues, including data storage and access policies, developers’ accountability, and data theft. She is concerned that data will be used for other purposes (e.g., criminal investigations), engendering users’ distrust.

Chapter 5 discusses whether contact-tracing apps are truly effective public health tools and if they exacerbate inequalities in societies. Landau cautions against measurement inaccuracies and low adoption rates. She provides examples that aid contact tracing while protecting users’ privacy, such as the UK NHS (National Health Service) COVID-19 app; this app scans NHS-supplied QR (Quick Response) codes at venues, then downloads hotspot identifiers that match the scanned codes to remind users if they have been to an infection hotspot. 

Chapter 6 advocates for a public policy discussion regarding the role of COVID-19 contact-tracing app in society. Landau makes policy recommendations in addition to safeguarding user data. First, COVID-19 should not trump other dimensions of well-being: if contact-tracing apps cause someone to isolate or lose a paycheck unnecessarily, they are not protecting all aspects of one’s well-being. Second, contact-tracing app usage must be a genuine choice; access to venues, transportation, or services should not be denied because someone refuses to use an app. Third, data collected should be used only for COVID-19 proximity checking; other uses should be prohibited. Fourth, contact-tracing apps should be evaluated before and during deployment in different communities. Fifth, app software should be open source to maintain transparency, and contact-tracing apps should undergo formal independent testing.

Although Landau covers contact-tracing apps of many countries, she does not directly comment on China’s health QR code, which is used for tracing citizens and denying venue and transportation access based on individuals’ risk status (*1*–*4*). In general, Landau cautions us against the surveillance state: should we normalize the idea of collecting proximity data via contact-tracing apps, governments could use the data to track political opponents and activists. She warns against accepting that it is normal for electronic devices to track our contacts. *People Count* reminds us that protecting citizens’ privacy and wellbeing are prerequisites for successful contact tracing, whether app-assisted or not.

## References

[1] Pan XB. Application of personal-oriented digital technology in preventing transmission of COVID-19, China. Ir J Med Sci. 2020;189:1145–6. 10.1007/s11845-020-02215-5 32219674 PMC7100464

[2] Yu A. Digital surveillance in post-coronavirus China: a feminist view on the price we pay. Gend Work Organ. 2020;27:774–7. [Epub ahead of print]. 10.1111/gwao.12471 32837009 PMC7280578

[3] Wang T, Jia F. The impact of health QR code system on older people in China during the COVID-19 outbreak. Age Ageing. 2021;50:55–6. 10.1093/ageing/afaa222 32990312 PMC7543282

[4] Liang F. COVID-19 and health code: how digital platforms tackle the pandemic in China. Soc Media Soc. 2020;6:2056305120947657. 10.1177/2056305120947657 34192023 PMC7424612

